# A novel conserved enhancer at zebrafish *zic3* and *zic6* loci drives neural expression

**DOI:** 10.1002/dvdy.69

**Published:** 2019-06-24

**Authors:** Rashid Minhas, Aleksandra Paterek, Maciej Łapiński, Michał Bazała, Vladimir Korzh, Cecilia L. Winata

**Affiliations:** ^1^ International Institute of Molecular and Cell Biology Warsaw Poland; ^2^ Randall Centre of Cell and Molecular Biophysics, Faculty of Life Sciences and Medicine King's College London London UK; ^3^ Department of Clinical Physiology Centre of Postgraduate Medical Education Warsaw Poland; ^4^ Max Planck Institute for Heart and Lung Research Bad Nauheim Germany

**Keywords:** *cis*‐regulation, conserved noncoding elements, development, gene regulation, habenula, transgenic zebrafish

## Abstract

**Background:**

Identifying enhancers and deciphering their putative roles represent a major step to better understand the mechanism of metazoan gene regulation, development, and the role of regulatory elements in disease. Comparative genomics and transgenic assays have been used with some success to identify critical regions that are involved in regulating the spatiotemporal expression of genes during embryogenesis.

**Results:**

We identified two novel tetrapod‐teleost conserved noncoding elements within the vicinity of the *zic3* and *zic6* loci in the zebrafish genome and demonstrated their ability to drive tissue‐specific expression in a transgenic zebrafish assay. The syntenic analysis and robust green fluorescent expression in the developing habenula in the stable transgenic line were correlated with known sites of endogenous *zic3* and *zic6* expression.

**Conclusion:**

This transgenic line that expresses green fluorescent protein in the habenula is a valuable resource for studying a specific population of cells in the zebrafish central nervous system. Our observations indicate that a genomic sequence that is conserved between humans and zebrafish acts as an enhancer that likely controls *zic3* and *zic6* expression.

## Key Findings

- Identified a novel enhancer near zebrafish *zic3/zic6* locus.
- The novel enhancer drives tissue‐specific expression in the habenula.
- Zebrafish transgenic line generated in this study can be a useful resource for studying development of habenula.

## 1 INTRODUCTION

Gene regulation is a highly complex process that requires the presence of intact coding sequences and the proper functioning of *cis*‐regulatory elements. The precise spatiotemporal activity of a gene involves the coordination of RNA polymerase, multiple transcription factors, and various *cis*‐regulatory elements, including enhancers, promoters, silencers, insulators, and locus control regions.^1^, ^2^ *cis*‐regulatory elements are often located upstream, downstream, within an intron, or hundreds of kilobases (kb) away from the genes they control.^2^, ^3^, ^4^ A gene might thus be surrounded by multiple *cis*‐regulatory elements, each of which combinatorially contributes to its overall spatial and temporal expression pattern. *cis*‐regulatory elements and gene expression also play a significant role in the evolution of vertebrate complexity and diversity during development.^5^, ^6^

The identification and functional characterization of *cis*‐regulatory elements are notable challenges in the postgenomic era and are needed to gain a better understanding of the language, syntax, and grammar that are encoded in regulatory DNA. Conserved noncoding elements (CNEs) from human to fish (evolutionary distance of 450 million years [Myr]) remain an important landmark to annotate the regulatory landscape around developmental regulators.^4^, ^7^, ^8^, ^9^ These noncoding genomic intervals can be highlighted by comparing the genomes of distantly related species. The CNEs that have been identified harbor multiple transcription factor binding sites (TFBSs). Mutations of these critical regions can alter gene expression and lead to anomalies and developmental defects.^1^, ^2^, ^7^, ^10^ Therefore, they are rarely lost during the evolution of distantly related species.^11^, ^12^

The vertebrate Zic family evolved by gene duplication events in higher vertebrates (*zic1*, *zic2*, *zic3, zic4*, *zic5*), with two additional *zic* family members in the zebrafish genome (z*ic2b* and *zic6* 13, 14). Mutations of *zic* genes lead to developmental anomalies of the cardiac, neural tube, skeleton, and muscles.^15^, ^16^ Zic genes are expressed in an overlapping fashion during early embryogenesis, particularly in the dorsal part of the neural tube and somites.^17^ However, differences in spatiotemporal expression patterns and different combinations of *zic* genes have been shown to be expressed in the limbs, eyes, and tail buds.^18^ The role of the Zic gene family has been extensively studied over the past two decades, but the regulatory elements that are responsible for the complex expression of Zic genes have not yet been elucidated in detail. The present study sought to identify novel functional enhancers around loci of the *zic3* and *zic6* genes.

Zebrafish *zic3* is paired with *zic6* in an opposite orientation in one of three extant loci that resulted from chromosomal duplication early in the evolution of vertebrates. Previously, two CNEs (E1 and E2) were reported to be nearby *zic3* and *zic6* loci, each of which was linked to both genes.^19^ The *zic3*‐associated enhancer was previously shown to drive expression in the neural tube and neural plate border.^19^ The present study employed comparative genomics and orthology mapping approaches to highlight putative enhancer regions around *zic3/zic6* and characterize their ability to drive the expression patterns of an enhanced green fluorescent protein (EGFP) reporter. Using UCSC Zv9/danRer7 genome assembly, we identified three noncoding genomic intervals that are close to *zic3/zic6* and have been under strong evolutionary pressure and conserved during the course of 450 Myr from human to fish. We identified and characterized a novel CNE that encompasses 453 bp and is a neural enhancer that regulates gene expression in the habenula during early embryogenesis that correlates with the expression pattern of *zic3* and *zic6*. Two other enhancers that we identified have partially overlapping sequences with previously reported *zic3/zic6*‐associated neural enhancers.^19^ Our results show that the novel intragenic CNE around the *zic3* and *zic6* loci acts as a neural‐specific enhancer and possibly drives the expression of *zic3* and *zic6*.

## 2 RESULTS

### 2.1 Tetrapod‐teleost CNEs maintain a conserved association with the *zic3* gene

**Figure 1:**
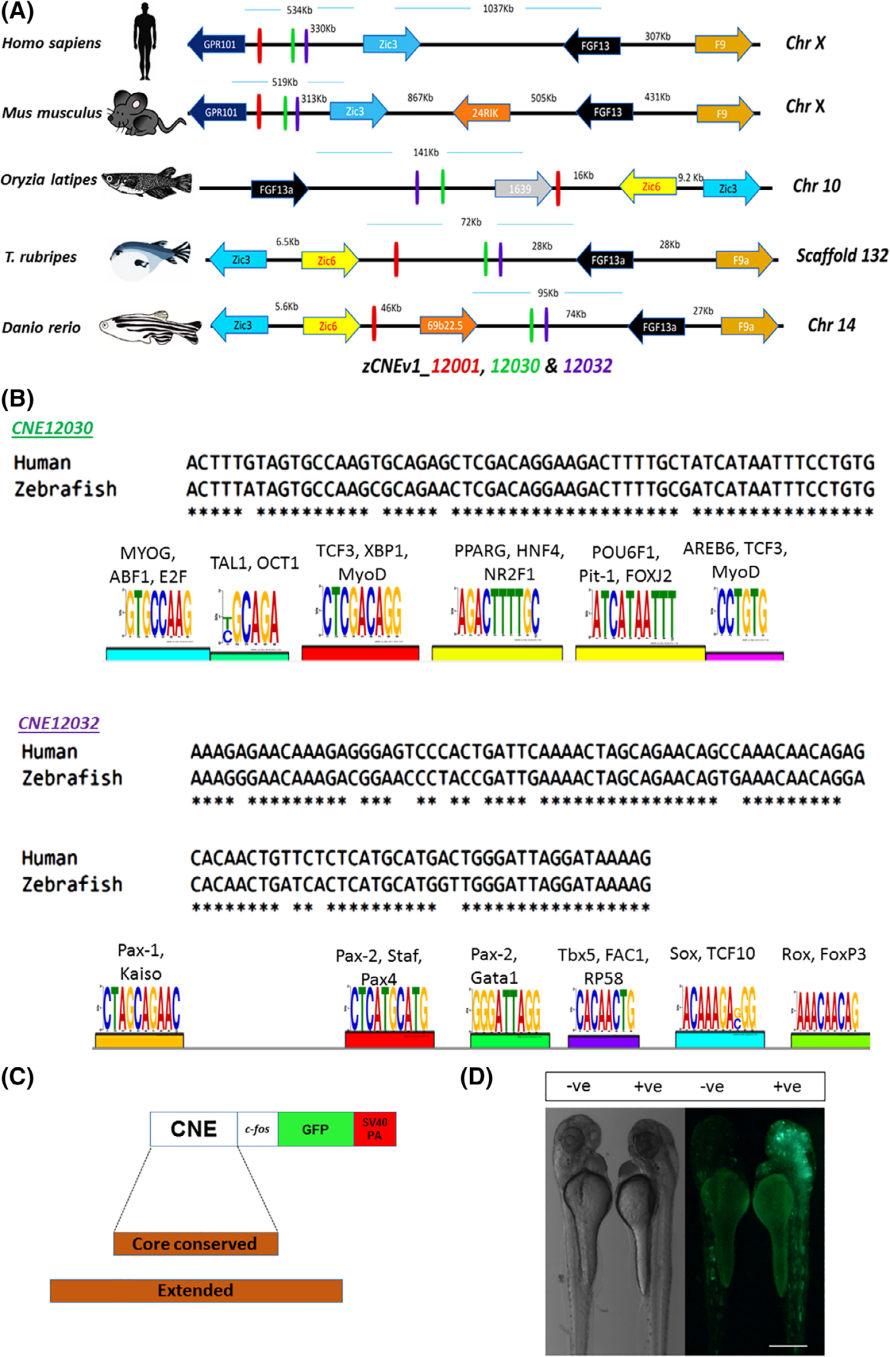
A, Comparative syntenic analysis of human, mouse, medaka, fugu, and zebrafish orthologous loci, depicting the conserved presence of two genes, *fgf13a* and *zic3*, in the nearest vicinity of three CNEs (red, green, and purple vertical lines). Genes are color‐coded. The arrow direction depicts the direction of gene transcription. The vertical line depicts the position of the CNE. The horizontal blue line depicts the scale. B, Human and zebrafish alignment of CNE12030 and CNE12032, highlighting the highly similar sequences between them and graphical representation of the transcription factor binding motif that was identified by MEME. Each of the motifs was further screened against the TRANSFAC library to mark binding sites for multiple developmentally important transcription factors. C, Schematic representation of the reporter gene cassette that was used for the in vivo characterization of CNEs. Brown vertical boxes represent different lengths of noncoding DNA that were selected for the transgenic assays. D, Embryos injected with Tol2–c‐*fos*–EGFP backbone without a CNE (left side embryos, negative) and with a CNE12032‐containing reporter construct (right side embryos, positive) at 48 hpf in the transient transgenic assay. Both pictures are lateral views with the dorsal side toward the right. Scale bar = 500 μm. Chr, chromosome; CNE, conserved noncoding element; EGFP, enhanced green fluorescent protein; MEME, multiple EM for motif elicitation

Multispecies alignment of the DNA sequence of the zebrafish *zic3/zic6* locus with orthologous genomic sequences from human, mouse, medaka, and fugu revealed three CNEs (Figure 1A) that maintain at least 80% sequence identity over a 60 bp window across all species (Table 1). For simplicity, these CNEs were named according to the zCNEv1 and UCSC genome browsers (CNE12001, CNE12030, and CNE12032). In the zebrafish genome, two of the CNEs (ie, CNE12030 [novel] and CNE12032) are located ∼93 kb upstream of *zic3*, and the third CNE (CNE12001) is located ∼28 kb upstream of *zic3* (Figure 1A, Table 1). To confirm whether these CNEs are truly associated with *zic3* and *zic6* in silico, a gene synteny was drawn for *zic3/zic6* and its flanking genes between tetrapod and teleost orthologous loci (Figure 1A). This comparison revealed that these CNEs maintained a syntenic relationship only with the *zic3* gene because the *zic6* gene is retained only in teleosts. These CNEs harbored motifs for important transcription factors, such as Tcf3, Oct1, Nr2f1, Pax, Pitx2, Tbx5, and Sox (Figure 1B, Table 2).

**Table 1:** Mammals‐teleost CNEs around zebrafish *zic3* locus selected for functional analysis in transgenic zebrafish assays

	Coordinates Chr14	Distance from *zic*3	Amplicon size	Human‐zebrafish conservation 80%; >60 bp	Primers	Transcription factor binding sites
CNE12001 Core	31 698 941‐31 699 074	~28 kb	134 bp	84% (134 bp)	5′‐GTAAATTCTTTTGAAGTGGAAC‐3′ 5′‐TATCATCATCAGTTTGGAATG‐3′	NRSF, ARF, v‐Myb ICSBP, ISGF‐3, IRF‐1, Pax‐6, Pax‐9, PDR3
CNE12001 Extended	31 698 785‐31 699 221	—	437 bp	—	5′‐TCCACCATTGTTTCGGCAACTC‐3′ 5′‐GGCGTAAAGCTTTGTAGACATA‐3′	—
CNE12030 Core	31 769 689‐31 769 749	~93 kb	118 bp	93% (61 bp)	5′‐ACTTTATAGTGCCAAGCGCAG‐3′ 5′‐CCACAGGAAATTATGATCGC‐3′	MYOG, ABF1, E2F, PPAR, HNF4, *NR2F1,POU6F1, Pit‐1*
CNE12030 Extended	31 769 414‐31 769 866	—	453 bp	—	5′‐TAAGGGGGTAAAGAACCTGA‐3′ 5′‐CTGTTCAAAGGGACAGTCATTG‐3′	—
CNE12032 Core	31 771 238‐31 771 338	~94 kb	101 bp	85% (101 bp)	5′‐AAAGGGAACAAAGACGGAACC‐3′ 5′‐CTTTTATCCTAATCCCAACC‐3′	PAX1, PAX2, TBX5, GATA1, FOXP3, TCF10, LEF1
CNE12032 Extended	31 771 104‐31 771 569	—	466 bp	—	5′‐CTCCCCTAATTACAGTCAGT‐3′ 5′‐CTGGTAGCAACAACCTCAGTC‐3′	—

*Note*: Location, coordinates (Ensembl 85: August 2016), amplicon size, and human‐zebrafish conservation score of selected subset of CNEs (functionally tested in this study) are indicated. In addition, the table also provides information about the forward and reverse primers used for amplification and cloning. All primers contained BamHI restriction site and an additional GC at 5′ end.

Abbreviation: CNE, conserved noncoding element.

**Table 2:** List and expression of transcription factors harboring conserved binding site in CNEs

TFs	Expression	Reference
Tal1	Diencephalon, hindbrain, optic tectum	Zhang and Rodaway (2007)^54^
Oct1	Brain	Mihaljevic et al. (2016)^55^
Xbp1	Neural plate, notochord, neural crest	Thisse (2004)^56^
pparg	Brain (qPCR)	Tseng et al. (2011)^57^
Nr2f1	Diencephalon, hindbrain neural keel, midbrain neural rod	Love and Prince (2012)^58^
Pou6f1	Central nervous system	Spaniol et al. (1996)^59^
Pax1	Notochord	Qiu et al. (2016)^60^
Pax2	Midbrain hindbrain boundary, midbrain, optic tectum	Kesavan et al. (2017)^61^
Tbx5	Central nervous system, eye, pectoral fin	He et al. (2011)^62^
Sox	Forebrain, hindbrain, hindbrain neural plate, lens	Okuda et al. (2006)^63^
TCF10/LEF1	Midbrain dorsal region	Lin and Lee (2016)^64^

*Note*: Subset of TFs expressed in the same region where CNEs reporters are expressed in zebrafish.

Abbreviations: CNE, conserved noncoding element; qPCR, quantitative PCR; TF, transcription factor.

### 2.2 Green fluorescent protein expression is driven by full‐length CNE12030 and CNE12032 in the transient assay

To investigate the potential role of these CNEs in detail, we initially performed transient transgenesis assays based on a *Tol2* vector with a *c‐fos* minimal promoter^20^ and tested two different lengths of each CNE (Figure 1C): (a) an extended or longer sequence and (b) a core conserved sequence between human and fish (Table 1). The injected embryos were screened at different developmental stages (∼26‐33 hours postfertilization [hpf], ∼48‐56 hpf, and ∼72 hpf) for GFP signals. Transient reporter gene activity was induced by the extended and core conserved regions of CNE12030 and CNE12032. However, both the extended and short lengths of CNE12001 failed to induce reproducible EGFP expression in the transient assays. We also injected a Tol2‐c*‐fos*‐EGFP backbone without a CNE in a transient assay as a negative control (Figure 1D). At both the 48 and 72 hpf stages, ∼69% (44/63) of the embryos that were injected with the Tol2‐c*‐fos*‐EGFP backbone without a CNE showed background expression in muscle cells as previously reported.^20^

**Figure 2:**
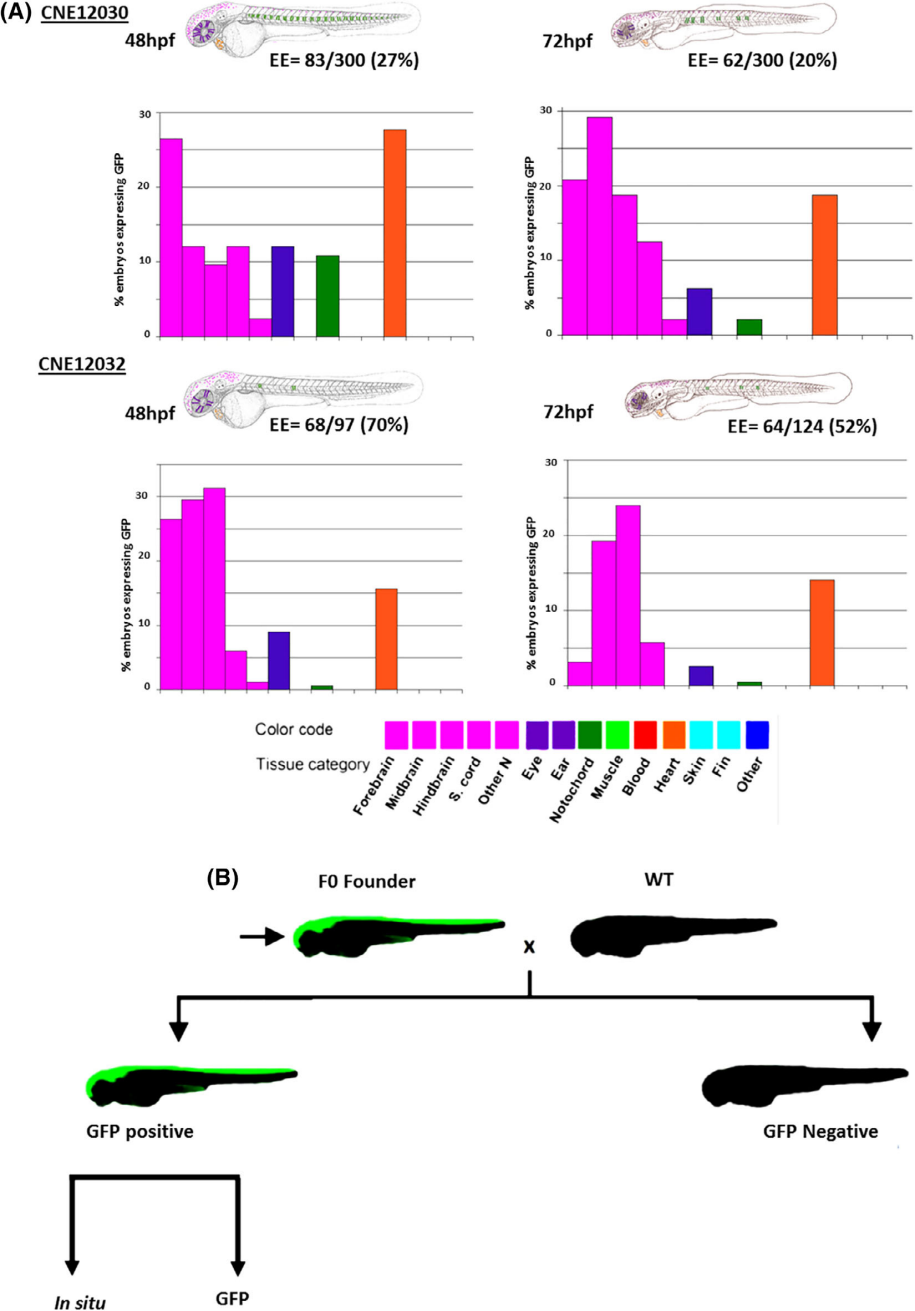
Sites of GFP signals that were recorded in zebrafish embryos that were transiently transfected with a full‐length construct. The reporter genes that were induced by individual CNEs (indicated by the name) are depicted in schematic representations of day 2 (48 hpf) or day 3 (72 hpf) embryos. Categories of a cell type that was positive for a given element are color‐coded, with a GFP‐expressing cell. The bar graphs (*y*‐axis) show the percentage of GFP‐expressing embryos that presented expression in each tissue category for a given element. The percentage of GFP‐expressing embryos per CNE is indicated beneath each schematic (EE = %). The bar graphs use the same color code as the schematics for each cell type. B, The injected fish were raised to adulthood and crossed with wild‐type fish to screen the germ‐line transmission of enhancers. Each GFP‐positive fish was further studied in situ to compliment the GFP signals and anti‐GFP to confirm GFP expression. CNE, conserved noncoding element; EE, GFP‐expressing embryos; GFP, green fluorescent protein; hpf, hours postfertilization

Based on our transient *Tol2* transgenesis results, we subcategorized GFP expression in several developmental domains as indicated in Figure 2A. The prominent domains that were observed in the transient assays for both CNEs were in the central nervous system (CNS; forebrain, midbrain, hindbrain), eyes, heart, and notochord.

### 2.3 CNE12030 drives GFP expression in the habenula

**Figure 3:**
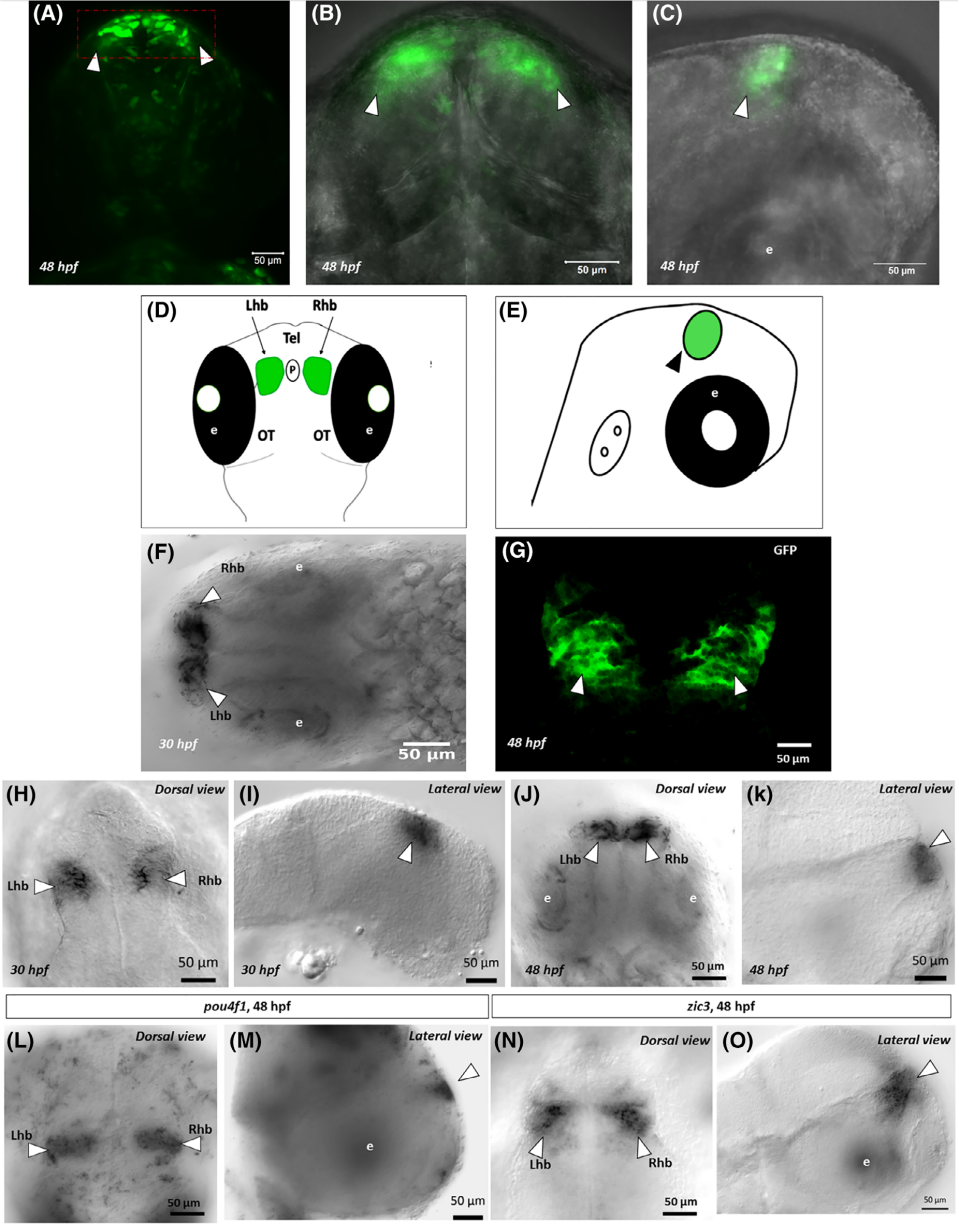
Green fluorescent protein expression in stable F1 line at 48 hpf. A, Green fluorescent protein expression in live embryos is indicated by arrowheads in the left and right habenula. B, The same GFP expression from the same embryo in a closer view. C, Green fluorescent protein expression from the lateral side. D and E, Schematics that show GFP expression in the habenula at 48 hpf in the dorsal and lateral views. F, RNA whole‐mount in situ hybridization using a probe against GFP, confirming its expression in the habenula at 30 hpf. G, Whole‐mount anti‐GFP immunohistochemistry, confirming GFP expression in the habenula at 48 hpf. H‐K, RNA whole‐mount in situ hybridization against GFP, showing GFP expression in the habenula at 30 hpf (H, I) and 48 hpf (J, K). L and M, RNA whole‐mount in situ hybridization using *pou4f1* probe labeling in the left and right habenula at 48 hpf. N and O, Whole‐mount in situ hybridization of *zic3* expression at 48 hpf. The photomicrographs in (H‐O) were taken using a Nomarski contrast microscope with a high‐sensitivity monochromatic camera (Zeiss AxioCam MRm). Scale bars = 50 μm. e, eye; GFP, green fluorescent protein; Lhb, left habenula; Rhb, right habenula

To generate stable transgenic lines, approximately 50 fish were injected with each CNE construct and raised to adulthood. After 3 months, founder fish were outcrossed with wild‐type fish for germline transmission screening in F1 (Figure 2B). Among the F1 offspring of founders that were injected with either CNE12030 or CN12032 core conserved regions, no reporter gene expression was observed. This suggested that no germline transmission occurred to the F1 generation. Therefore, we were unable to validate the activity of these shorter versions of the CNE. Germline transmission was identified in two F0 females (4%) that carried the longer form of CNE12030. The expression domain for both founders was the habenula, with 15 to 20 GFP‐positive embryos per 100 F1 embryos. Comparisons of GFP signals and intensity revealed that both founders had negligible differences in expression patterns. However, one had higher levels of GFP expression in the habenula (Figure 3A‐G). GFP expression in the habenula in stable transgenics persisted from early embryogenesis (ie, day 1‐3). To characterize other weaker signals in more detail, RNA whole‐mount in situ hybridization was performed using an antisense probe against GFP (Figure 3F,H‐K). Whole‐mount anti‐GFP immunohistochemistry was also performed to search for domains of GFP expression other than the habenula, but no other additional domains were identified (Figure 3G). GFP transcripts were detected in the same region where GFP signals were present at 30 hpf (Figure 3H,I) and 48 hpf (Figure 3J,K). To confirm that the structure that expressed GFP was indeed the habenula, RNA whole‐mount in situ hybridization was performed using an antisense probe against a known habenula marker, *pou4f1* 21; Figure 3L,M). Whole‐mount in situ hybridization for *pou4f1* revealed expression in the same territory of the brain as that of GFP, thus confirming that GFP‐expressing cells were in the habenula (Figure 3H‐M). Moreover, RNA in situ hybridization using an antisense probe against *zic3* (Figure 3N,O) showed that CNE12030 transgene expression coincided with known sites of *zic3* expression.^22^, ^23^ Therefore, the extended version of CNE12030 was shown to drive expression in the habenula, which coincided with the *zic3* expression domain.

### 2.4 CNE12032 drives expression predominantly in the optic tectum, olfactory pit, spinal cord, fin, and heart

**Figure 4:**
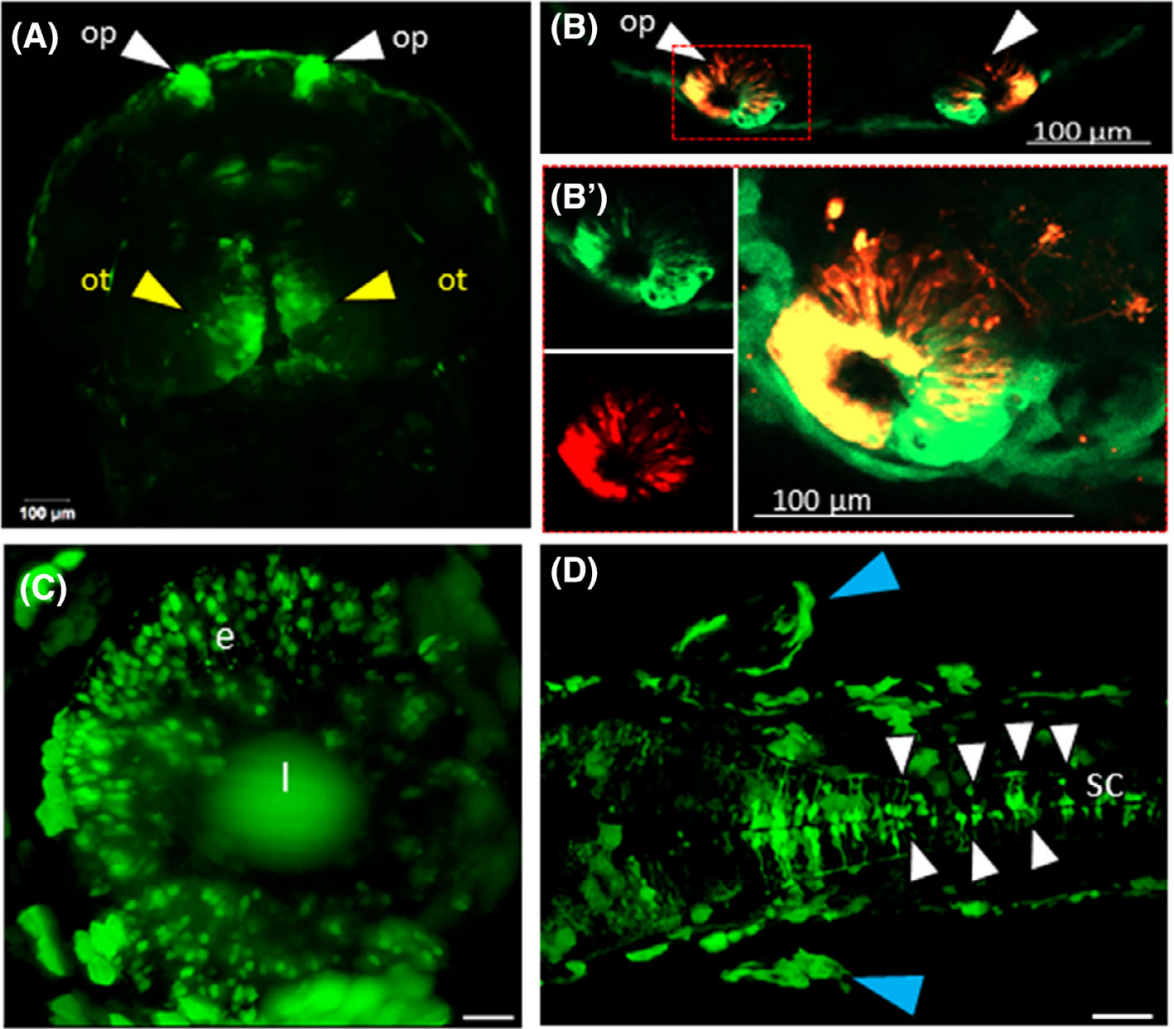
Robust expression pattern driven by CNE12032. Live fluorescent images of CNE12032 stable transgenic zebrafish embryo on day 2. Green‐fluorescent protein‐expressing regions in live embryos are shown, indicated by arrowheads. A, Confocal Z‐stack images of the dorsal view. The white arrowhead indicates GFP‐expressing cells in the olfactory pit. The yellow arrows show the optic tectum. B, Merged channel, highlighting colocalized DiI with EGFP in olfactory sensory neurons. B′, DiI fluorescence in olfactory neurons in the same embryo in a closer view. C, Light‐sheet Z‐stack images in the lateral view, anterior to the left. The white arrow indicates GFP signals in the eye. D, Light‐sheet Z‐stack images in the dorsal view, anterior to the left. The white arrowheads indicate GFP signals in the spinal cord. The blue arrowheads indicate GFP signals in the pectoral fin. Scale bars = 20 μm in (C) and 50 μm in (D). CNE, conserved noncoding element; e, eye; EGFP, enhanced green fluorescent protein; GFP, green fluorescent protein; l, lens; op, olfactory pit; ot, optic tectum; pf, pectoral fin; sc, spinal cord

CNE12032 that is located ∼72 kb downstream of *fgf13a* and ∼95 kb downstream of *zic3* is conserved from human to fish. The 466‐bp genomic segment that spanned the human‐fish conserved core sequence (101 bp) drove GFP expression in founder fish, although we identified only one male individual with germline transmission (ie, 2% of the total number of fish that were injected). However, GFP expression was robust and widespread in several brain regions compared with the other CNE. This expression pattern was similar to previously reported domains of this CNE in the neural tube,^19^ with some additional domains that have not been reported previously, including olfactory neurons, the eyes, the pectoral fin, and the heart (Figure 4, Video S1). To confirm GFP expression in olfactory neurons, cells that expressed GFP in olfactory receptor neurons were stained with the lipophilic fluorescent membrane tracer dye DiI and simultaneously imaged for GFP and dye fluorescence using a confocal microscope. We observed the colocalization of GFP and DiI fluorescence in olfactory neurons (Figure 4B). GFP expression in the optic tectum, eyes, pectoral fin, olfactory pit, and heart persisted from 24 to 72 hpf. These results suggest that this enhancer may be responsible for driving gene expression not only in the neural tube but also in the pectoral fin, the heart, and sensory organs. This expression pattern that was driven by CNE12032 corresponded to distinct domains of *zic3* expression compared to that driven by CNE12030.

Overall, this in vivo analysis of stable zebrafish transgenics identified two different enhancers in the vicinity of *zic3*/*zic6* loci in zebrafish with different tissue specificity. These two CNEs may thus drive expression in a subset of tissues that express Zic3.

## 3 DISCUSSION

The Zic gene family contributes to the patterning and growth of many organs, including the CNS, the heart, retinal ganglion cells, somites, and limbs, during vertebrate embryogenesis. Among the Zic family, Zic3 plays a key role in development and disease. Studies have shown that the loss of Zic3 function leads to congenital developmental defects, including heterotaxy and multiple types of congenital heart defects.^24^, ^25^ Comparative genomics‐based approaches have emerged as reliable tools for predicting noncoding genomic features that harbor transcriptional regulatory elements, even in the absence of knowledge about the detailed characteristics of individual *cis*‐regulatory elements.^3^, ^4^, ^26^, ^27^, ^28^ Two critical steps have been suggested to be necessary to ascertain a functional CNE: (a) carefully selecting the species that are compared and (b) choosing the appropriate length of genomic intervals.^29^ Depending on the alignment tools, different levels of stringency are applied to identify a number of putative enhancers, without an a priori biological rationale.^30^ Conventional comparative criteria, such as 70% sequence identity over at least 100 bp between human and mouse, have disadvantages because these two species share a relatively short evolutionary distance (∼65 Myr). The most obvious solution to identify the functional module in the genome is to compare more distantly related species, such as humans and fish (∼450 Myr), using suitable identification criteria. The functional validation of CNEs should include additional steps beyond steps (a) and (b) above, such as (c) analyzing the ability of the CNE to drive expression in transient transgenics and (d) identifying the ability of the CNE to drive expression in stable transgenics. Furthermore, random integration of the reporter construct (positional effect) and the lack of a positive control for transgenesis pose challenges to defining the efficacy of integration events in F0 embryos and in nonexpressing F1 embryos.^31^ In this case, positive stable expression in F1, together with mapping of the insertion site wherever possible, is necessary to validate the results of enhancer activity assays.

In the present study, we initially selected 32 CNEs that were predicted by the Bejerano group and available with the custom track Bej zCNEv1 in the UCSC genome browser in the ∼158 kb intragenic region between *zic3* and *fgf13a*.^32^ Using the relatively stringent criteria of 80% sequence identity within the 60‐bp window, we ascertained three of 32 elements that are highly conserved from human to fish. The gene environment of the loci that harbored the subset of CNEs was calculated further to associate the selected CNEs with their target gene body using an orthology mapping approach. Zebrafish *zic3* and *zic6* loci have been compared with orthologous loci from genomic sequences of *Homo sapiens* (mammal), *Mus musculus* (rodent), *Oryzias latipes* (teleost fish), and *Takifugu rubripes* (teleost fish; Figure 1). In teleosts, CNE12001, CNE12030, and CNE12032 maintained a conserved physical linkage with *fgf13a*, *zic6*, and *zic3*. Closer inspection of our syntenic analysis and previously reported studies showed that *Zic6* was deleted during mammalian evolution, whereas *Zic3* maintained its association with these CNEs.^33^

Three lines of evidence corroborate the CNEs that were identified as *zic3*/*zic6* enhancers in the present study. First, the in vivo reporter expression pattern that was driven by CNE12030 and CNE12032 coincided with the pattern that was previously reported for *zic3* in the CNS.^13^, ^33^ Second, the presence of conserved binding sites for recognized developmental regulators based on rigorous criteria in both zebrafish‐human tracks suggested that these CNEs may be regulated by transcription factors (Table 2) that are coregulated with *zic3* during early embryonic development of the CNS, such as members of the Wnt and Nodal pathways.^34^, ^35^ Third, a survey of expression patterns of all genes within 1.5 Mb up‐ and downstream of the *zic3*/*zic6* cluster did not identify other candidates with clear expression in the habenula (ZFIN database), strongly suggesting that *zic3* and *zic6* are the only obvious targets within the vicinity of these enhancers.

The habenula is part of a highly conserved limbic system conduction pathway in vertebrates and has been shown to play a crucial role in a diverse set of behavioral systems, including brain stimulation, endocrine function, mating, ingestion, fear responses, and olfaction.^36^, ^37^, ^38^ The habenula in zebrafish is subdivided into the dorsal and ventral habenula, which are homologous to lateral and medial parts in mammals.^39^ Within the CNS, *zic3* is known to be expressed in a restricted manner during neurulation.^17^ In the present study, CNE12030 was shown to drive GFP expression in the habenula (Figure 3A‐C,H‐K) where *zic3* is expressed (Figure 3N,O). Additionally, the habenula‐specific marker *pou4f1* validated that the CNE12030‐driven expression domain represents the right and left habenula. Altogether, for *zic3* and *pou4f1*, the novel CNE12030 might play important roles in the development of the habenula. This possibility could be confirmed by deleting this CNE or mutating binding sites for multiple transcription factors. The zebrafish is an ideal model for elucidating neurocircuitry mechanisms that underlie animal behavior.^40^ Further investigations of this transgenic line may open new avenues for studying the vertebrate brain limbic system. Moreover, this novel CNE will further our understanding of the genetic basis of human birth defects that cannot be attributed to a mutation in the coding sequence of Zic3.

Garnett et al^19^ previously showed that the enhancer E2 that is evolutionarily conserved between humans and fish autonomously controlled individual aspects of *zic3* expression in the developing neural tube in zebrafish embryos. CNE12032 is located ∼74 kb downstream of *fgf13a* and ∼93 kb upstream of *zic3*, which overlaps by an additional 112 bp with E2. Interestingly, in the stable transgenic line, the overall expression pattern that was driven by CNE12032 was similar to E2, particularly in the CNS, with some additional domains that were not previously reported. These novel domains of prominent GFP signals that were observed at 48 and 72 hpf included cardiac cells, the pectoral fin, and olfactory sensory neurons. This largely fits with the known expression patterns of the Zic family in mice, chick embryos, and zebrafish, which are expressed in a partially overlapping manner within the neural tube, somites, and other ectoderm‐ and mesoderm‐derived structures in the future head and trunk.^14^, ^17^, ^41^

The transgenic line ET33‐mi59B carries an enhancer trap that inserted 155 kb upstream of *zic3* and 140 kb downstream of *zic6* and exhibits GFP expression in the cardiac conduction system of the heart.^42^ However, none of our tested CNEs that were located ∼93 kb upstream of *zic3* drove GFP expression in cardiac conduction system cells at 48 to 72 hpf. The enhancer that is responsible for cardiac conduction system‐specific expression may not be conserved from humans to fish as many enhancers were previously reported to be nonconserved.^43^ Alternatively, additional flanking sequences that were omitted in the present analysis may contribute to a more comprehensive enhancer element that may control specific expression in an additional subset of tissues.^29^ This is a particularly interesting possibility because of the presence of the *fgf13a* gene, which has been implicated in heart development,^42^ adjacent to the *zic3* locus (167 kb upstream).

## 4 CONCLUSION

The present study used a comparative genomics approach to highlight putative enhancers around the *zic3* and *zic6* loci. Like other members of Zic family genes, *zic3* and *zic6* might share common regulatory elements. The novel enhancer CNE12030 drove GFP expression in the habenula, which is a key part of the CNS (diencephalon) and is highly conserved from teleost fish to mammals. The present findings suggest that the newly identified CNE12030 may be involved in habenula development and spatially control *zic3* expression. This transgenic line can be a useful tool for studying zebrafish habenula development and function.

## 5 EXPERIMENTAL PROCEDURES

### 5.1 Selection of putative *cis*‐regulatory elements around *zic3*‐*zic6* for functional analysis

CNEs around *zic3* and *zic6* loci were selected for functional studies from annotated regions^32^ that are available at the UCSC Zv9/danRer7 genome browser (http://genome.ucsc.edu/) with a custom track of Bej zCNEv1. The subset of predicted CNEs was further subjected to BLASTN with human, mouse, medaka, and fugu genomes using the Ensembl genome browser, applying stringent conservation criteria of at least a 60 bp length and 80% sequence identity. CNEs that were only conserved in teleost fish and did not meet our selection criteria were discarded. Three CNEs upstream of *zic3* and downstream of *zic6* (CNE12001, CNE12030, and CNE12032) were selected for further functional studies.

### 5.2 In silico analysis of CNEs from mammals to teleost fish to identify conserved putative TFBSs

To identify putative conserved TFBSs for each CNE, orthologous sequences from mammals to teleost fish were retrieved from the Ensembl genome browser using a BLASTN‐based similarity search as previously described.^3^ Each of the CNEs with orthologous sequences was evaluated using the Multiple Em for Motif Elicitation (MEME) algorithm.^44^ According to the expected length of DNA sequences that are recognized by transcription factors, the criteria for the minimum length was set to 6 to 12 bp.^45^, ^46^ The identified motifs of each CNE were further characterized using the STAMP online tool^47^ to determine known transcription factors against the TRANSFAC (v. 11.3) database.^48^ The gene expression patterns of each of the specified transcription factors were checked in the ZFIN database (https://zfin.org) and are listed in Table 2.

### 5.3 Zebrafish transgenic assays and screening

Wild‐type and transgenic zebrafish were maintained in the zebrafish facility of the International Institute of Molecular and Cell Biology in Warsaw (license no. PL14656251) according to standard procedures and ethical practices. Embryos were grown in embryo medium at 28°C and staged according to standard morphological criteria.^49^ The transgenic zebrafish experiments were performed according to standard protocols that were established by the Polish Laboratory Animal Science Association. Putative enhancer regions were amplified by polymerase chain reaction (PCR) from wild‐type zebrafish genomic DNA using primers with the BamHI site (Table 1). The PCR products were then purified using the MN DNA purification kit (Macherey‐Nagel) according to the manufacturer's instructions and subsequently cloned into the BamHI site of a reporter system that consisted of EGFP under the control of the mouse c*‐fos* minimal promoter and *Tol2* sites. Each of the plasmid constructs that contained the CNEs was then sequenced to control for the presence of any point mutations that were generated during PCR amplification. The reporter constructs that contained the Tol2‐c‐*fos*‐EGFP promoter‐reporter cassette^50^ were combined with 0.5% phenol red (Sigma), which was used as a tracer dye as described previously.^3^, ^51^ Injection mix (1 nL) was injected into 1 to 2 cell‐stage zebrafish embryos using the PV820 pressure injection system (WPI) under an SZX16 stereo microscope (Olympus). The Tol2‐c‐*fos*‐EGFP plasmid without CNEs was also injected as a negative control. The injected embryos were raised at 28.5°C in 1X embryo medium. The zebrafish embryos were dechorionated manually using fine forceps at 2 days postfertilization (dpf) and anesthetized using tricaine.^52^ The transient transgenic embryos were screened for GFP signals under an M165 FC inverted fluorescent microscope (Leica). Imaging was performed using the Leica application suite (v. 4.0) and Zeiss Axio Imager 2 using Axiocam MRm. The schematics for the location and tissue‐specific expression of each CNE were generated using Adobe Photoshop software. Approximately 50 embryos with GFP signals were raised to adulthood to screen germ line transmission.

### 5.4 Imaging using light‐sheet fluorescence and confocal microscopy

Embryos were anesthetized at the desired developmental stages and embedded in a ∼1 mm inner‐diameter glass capillary that was filled with 1.5% low‐melting agarose (LMA) in embryo medium (0.03% Instant Ocean salt into double‐distilled water). After the complete polymerization of agarose, the capillary was inserted in the sample holder and placed in the microscope chamber that was filled with embryo medium (0.02% tricaine), and the embryo was pushed out of the capillary for imaging. The temperature of the sample chamber was maintained at 28°C during imaging. Image acquisition was performed using a Zeiss Z.1 light‐sheet microscope with a W Plan‐Apochromat 20×/1.0 objective. Z‐stacks (3.83 μm thickness, 60 ms exposure time) were saved in LSM format and then processed using ZEN software (Zeiss). For each Z‐stack, maximum‐intensity projections were generated. For confocal microscopy, embryos were mounted in 1.5% LMA in glass‐bottom dishes after the complete polymerization of LMA.

### 5.5 Whole‐mount zebrafish larvae immunofluorescence

Zebrafish embryos were fixed with 4% paraformaldehyde at 4°C overnight. Embryos were permeabilized in methanol, rehydrated in a series of MeOH/PBST (Phosphate Buffer Saline with Tween 20) concentrations (75%, 50%, and 25%), and washed with 1X PBST. Treatment with Proteinase K (10 μg/mL) for 30 minutes was followed by postfixation in 4% paraformaldehyde for 30 minutes and several washes in PBST. Embryos were blocked with blocking solution (10% sheep serum and 1% dimethylsulfoxide [DMSO] in PBST) for 4 hours to reduce nonspecific antibody binding and then incubated overnight at 4°C with rabbit polyclonal anti‐GFP antibody (Gentex) at a dilution of 1:500. After incubation, several washes were performed, and the primary antibody was detected using Alexa Fluor 488 Donkey anti‐Rabbit IgG secondary antibody (Invitrogen) at a dilution of 1:200. Embryos were stored in PBST at 4°C. Images were taken using a Zeiss LSM 800 microscope after mounting in 1.5% LMA.

### 5.6 Whole‐mount in situ hybridization

Whole‐mount in situ hybridization of zebrafish embryos at 30 hpf was performed using a digoxigenin‐labeled antisense RNA probe. The *pou4f1* cLone was provided by Steve Wilson UCL, UK. The probe that was specific for *zic3* was synthesized from 1990 bp fragment cDNA using the DIG‐RNA labeling kit (Roche) according to the manufacturer's instructions. Whole‐mount in situ hybridization was performed as described previously^53^ with minor modifications. After rehydration, permeabilization, and blocking, the RNA probe was hybridized to target mRNA overnight at 68°C. Anti‐digoxigenin‐AP Fab fragments (Roche) were used for target recognition. The color reaction was performed using NBT/BCIP Solution (Roche).

### 5.7 DiI staining to label olfactory pits

DiI stain (1 μL) was dissolved in 100% DMSO (final concentration of 2.5 mg per 1 mL of DMSO) and used for 1 mL of embryo media. The embryos were then incubated for 15 to 30 minutes at 28°C. To remove residual DiI stain, the embryos were washed three times with embryo medium. Olfactory pits were observed with red fluorescence at an excitation wavelength of 549 nm and emission wavelength of 565 nm.

## CONFLICT OF INTEREST

The authors declare no potential conflict of interest.

## AUTHOR CONTRIBUTIONS

R.M., V.K., and C.L.W. conceived the project and designed the experiments. Computational analysis was performed by R.M., A.P., and M.L., and M.B. performed experiments. The manuscript was written by R.M., V.K., and C.L.W.

## Supporting information


**Video S1**
